# Maternal undernutrition and offspring sex determine birth-weight, postnatal development and meat characteristics in traditional swine breeds

**DOI:** 10.1186/s40104-018-0240-6

**Published:** 2018-03-19

**Authors:** M. Vázquez-Gómez, C. García-Contreras, L. Torres-Rovira, S. Astiz, C. Óvilo, A. González-Bulnes, B. Isabel

**Affiliations:** 1 Faculty of Veterinary Medicine, UCM, Madrid, Spain; 2 INIA, Madrid, Spain

**Keywords:** Carcass quality, Fatty acids, Feed restriction, Growth, Iberian pigs, Low birth-weight, Malnutrition

## Abstract

**Background:**

The aim of this study was to determine how maternal undernutrition during pregnancy and offspring birth-weight can affect the postnatal development of offspring under farm conditions, which may lead to consequences in its meat and carcass quality. The current study involved a total of 80 litters from Iberian sows fed a diet fulfilling daily requirements (*n* = 47; control) or providing 70% daily requirements (*n* = 33; underfed) from d 38 to d 90 of gestation when fetal tissue development begins. After birth, piglets born live were classified as low birth-weight (LBW; < 1 kg) and normal birth-weight (NBW; ≥1 kg). During the growing phase, 240 control and 230 underfed pigs (50% males and females) distributed by BW category and sex were studied until the slaughter.

**Results:**

At birth and weaning, there were significant differences in all morphological measures and weight between NBW and LBW piglets as expected (*P* < 0.0005), but few effects of the gestational feed restriction. During the growing phase, NBW pigs continued with higher weight than LBW pigs on all the days of evaluation (*P* < 0.05), even though control-LBW-females and LBW-males showed a catch-up growth. However, underfed pigs showed slower growth and higher feed conversion ratio than control pigs (*P* < 0.0001) at 215 days old. Moreover, the average daily weight gain (ADWG) for the overall period was greater for NBW, male and control pigs than for their LBW, female and underfed pigs (*P* < 0.0001, *P*< 0.0005 and *P*< 0.05, respectively) and NBW pigs were slaughtered at a younger age than LBW pigs (*P* < 0.0001). After slaughtering, control pigs also had higher carcass yield and backfat depth than underfed pigs (*P* < 0.0005) and the maternal nutritional effect caused main changes in the polar lipid fraction of liver and loin. The fatty acid composition of loin in control pigs had higher C18:1n-9 and n-3 FA concentrations, as well as lower ∑n-6/∑n-3 ratio, than in underfed pigs (*P* < 0.005).

**Conclusions:**

In brief, results showed that the effects of maternal nutritional restriction appeared and increased with offspring age, causing worse developmental patterns for underfed pigs than for control pigs.

**Electronic supplementary material:**

The online version of this article (10.1186/s40104-018-0240-6) contains supplementary material, which is available to authorized users.

## Background

Modern swine production (mainly located in Europe, USA, Brazil, China and other countries from Southeast Asia) is based on highly-selected genotypes (mostly originated from Landrace, Large-White and Pietrain breeds) reared in large farms generating value-for-money fresh pork products. In addition to this intensive production, there is also a local European industry (currently spreading to some countries of Asia and America) which is based on the use of traditional breeds for the elaboration of high-quality dry-cured products (ham, cured-loin, spiced-sausage, and salami). The most representative traditional breeds are the Iberian (Spain and Portugal) and Mangalica (Hungary) pigs, but there are other breeds reared in France, Germany, and Italy. These pigs have a high potential for fat accumulation and the content and composition of intramuscular fat give pork products their smooth texture and outstanding taste. In practice, these individuals are reared either as purebred or crossbred with Duroc boars for improving meat yields. Currently, the increasing demand for dry-cured ham and other gourmet products is transforming the traditional extensive farming of these pigs into more intensive systems, with management practices directly implemented from modern breeds; mainly at the reproductive (aiming to increase the number of piglets/litter) and nutritional levels (imitating feeding strategies).

The number of piglets per litter (prolificacy) is determined by the number of ovulations and, afterward, by the number of developing conceptuses. However, studies in modern breeds have shown that higher prolificacy compromises the proper fetal development, due to competition among littermates for the limited space available in the uterus for implantation and adequate placental development [1]; the so-called uterine capacity. Inadequacies of placental development affect the functionality of the organ and, therefore, the supply of oxygen and nutrients to the fetus. The consequence is the impairment of fetal growth (a process known as intrauterine growth restriction; IUGR), leading to low birth-weight (LBW) neonates [2]. The greater number of piglets in the litter, the higher incidence of LBW individuals [3, 4]. Traditional breeds like Mangalica and Iberian pig are characterized by a lower prolificacy and a smaller uterus (a lower uterine capacity) than prolific sows [5, 6]. Therefore, small increases in prolificacy are directly related to increases in fetal losses and LBW incidence [7]. The implementation of feeding strategies based on maternal feed restriction for lowering production costs in modern breeds has also been found to be related to a higher rate of IUGR processes and LBW piglets [2, 8]. The same has been found in the Iberian pig [9, 10].

The pork market, both in modern and traditional systems, requires batches of pigs with uniform weight, carcass conformation, and meat composition; even more in the case of high-quality products. Hence, LBW pigs reduce the value of farm products. Firstly, the appearance of LBW pigs causes a lack of homogeneity within litters and feedlots. Second, LBW piglets show higher morbidity and mortality, and lower growth potential, lower feed efficiency and lower meat yield than their normal birth-weight (NBW) littermates [11–13]. Moreover, LBW piglets may modify their physiology and metabolism by prenatal programming in response to the inadequate intrauterine environment, either of maternal or placental origin [14, 15]. After birth, these individuals are predisposed to excess adiposity as an adaptive mechanism for energy storing and survival in the inadequate postnatal environment expected, so carcass yields and meat quality would be affected.

In summary, these differences cause variability in carcass conformation and meat quality among pigs in the same litter and feedlot, which is negative for commercialization of the products. Such effects may be even more evident and negative in traditional swine. Firstly, due to the intrinsic developmental patterns of the breeds, in which IUGR individuals are even more prone to fat deposition after prenatal restriction [16–18]. Moreover, Iberian sows are highly predisposed to the mobilization of fat depots, insulin resistance and dyslipidemia in the case of undernutrition and maternal dyslipidemia affects placental efficiency and fetus metabolism and growth [7], increasing incidence and consequences of LBW offspring. Finally, traditional systems are based on longer productive cycles, where animals are slaughtered at a higher weight and therefore older age than lean commercial breeds (140–160 kg live-weight at 10–12 months old). Consequently, homogeneity of feedlots is affected due to over-time increased growth differences between LBW and NBW pigs with differences of several weeks or even months for reaching the target weight for the slaughterhouse.

Despite these considerations, there are scarce data regarding incidence and consequences on postnatal development and meat characteristics of LBW offspring in Iberian pigs and similar breeds reared in commercial farms. Hence, the aim of this study was to determine, for Iberian pigs under farm conditions, the effects of birth-weight and strategies based on maternal feed restriction on at-birth phenotypic characteristics, postnatal development and metabolism, and meat and carcass quality.

## Methods

### Animals and handling

Animal management was performed in agreement with the Spanish Policy for Animal Protection RD53/2013, which meets the European Union Directive 2010/63/UE about the protection of animals used in research. The experimental procedures were assessed and approved (report CEEA 2012/036) by the INIA Committee of Ethics in Animal Research (the Institutional Animal Care and Use Committee). All the animals (sows and piglets) were housed indoors, with a controlled temperature of around 22 °C, at the farm of Ibéricos de Arauzo 2004 S.L. (Zorita de la Frontera, Salamanca), either at individual (sows) or collective pens (offspring).

**Fig. 1:**
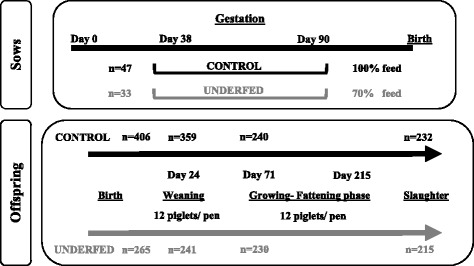
Schematic representation of study design

The design of the study is shown in Fig. 1. Each of the steps of the procedure is detailed in this section. The study involved a total of 80 litters from Iberian sows inseminated with Duroc cooled semen (PIC, Genus plc, UK). The sows were of 3^rd^ and 4^th^ parity and were evenly divided into two experimental groups. Forty-seven sows (CONTROL group) were individually fed with a standard grain-based diet by chip identification throughout the entire pregnancy (composition at Additional File 1: Table S1), calculated for fulfilling daily maintenance requirements for Iberian breed [19]. Thirty-three sows (UNDERFED group) were fed the same diet, but the amount was adjusted to fulfill only 70% of their daily maintenance requirements from d 38 to 90 of pregnancy. At farrowing, the numbers of total piglets born and born live were determined for each sow and, immediately, all the live piglets were examined for sex determination, weight recording and individual identification with earrings. Piglets were classified by their birth-weight (BW) as low and normal BW (LBW and NBW, respectively). The criterion for LBW was defined as a BW lesser than one standard deviation of the mean value of the control littermates (BW < 1 kg [20, 21]). Piglets remained with the mothers until weaning, after within-treatment fostering for equaling the number of piglets among sows. Offspring were managed following the regular practices of the farm.

The study involved 406 and 265 piglets born live from control and underfed pregnancies, respectively, with similar percentages of females and males (overall: 49.2% females vs. 50.8% males). At weaning, performed around 24 d of age, piglets were grouped by maternal diet, BW, and sex in pens of 12 piglets. Postnatal development was assessed during the transition phase (25 to 70 d of age) in a total of 359 control (20 LBW and 166 NBW females, and 21 LBW and 152 NBW males) and 241 underfed piglets (14 LBW and 106 NBW females, and. 15 LBW and 106 NBW males). Afterward, 240 control (16 LBW and 104 NBW pigs by each sex) and 230 underfed (12 LBW and 103 NBW pigs by each sex) pigs were randomly selected for studying development during the growing and fattening phases (from 71 days old to the slaughter). Finally, a total of 232 control (115 females and 117 males) and 215 (107 females and 108 males) underfed pigs were slaughtered after reaching the minimum weight established by the Spanish Policy for Iberian Products (115 kg of carcass weight).

### Assessment of postnatal development and yield indexes

At birth and weaning, all the piglets were weighed and measured for occipito-nasal length, biparietal diameter, trunk length, maximum thoracic diameter and abdominal and thoracic circumferences. Animals were weighed again at average ages of 110, 150, 180 and 215 d, and final slaughter-weight and age were recorded at the slaughterhouse. These values were used for calculating average daily weight gain (ADWG) and feed conversion ratio (FCR) in four intermediate periods of age: 25–110, 111–150, 151–180 and 181–215 d of age (each period is named as its last day). The ADWG values for suckling phase and whole life were also calculated. The formula of ADWG was [(final weight-initial weight)/number of days], while FCR was determined as [daily block feed intake mean/ADWG of the period]. Furthermore, backfat depth (total and divided in outer and inner layers) and loin diameter were determined at the P2 point at the level of the head of the last rib, at weaning and at 215 days old by using a SonoSite S-Series ultrasound machine equipped with a multifrequency lineal array probe (5–8 MHz; SonoSite Inc., USA).

### Assessment of carcass features and tissue sampling at slaughter

Total weight, total length and backfat thickness were recorded for all the carcasses immediately after slaughter. Carcass length was determined from the posterior edge of the *symphysis pubica* to the anterior edge of the first rib, while backfat thickness was measured in the midline of the carcass and at the level of the last rib (skin not included). In each pig, the value of carcass weight was used to determine carcass yield by using the formula [carcass weight/body weight], expressed as a percentage.

Immediately after slaughter, samples of subcutaneous fat and *longissimus dorsi* (LD) muscle were drawn at the midline of the carcass and collected from the level of the last rib (at the point used for measuring backfat depth) and samples of hepatic tissue were obtained from the right lobe of the liver. All these samples were immediately packaged in individual bags and stored at − 20 °C until analyzed for fatty acids (FA) composition analysis. A second sample of LD was used for analyzing drip-loss and moisture. In brief, at the same day of sampling, drip loss of LD muscle was determined by using the method described by Calvo et al. [22], while moisture was determined by drying samples at 110 °C [23].

### Evaluation of plasma indexes of carbohydrates and lipids metabolism

At 215 days old, a blood sample for each pig was drawn from the orbital sinus by using 5 mL sterile heparin vacuum tubes (Vacutainer Systems Europe, France). At slaughter, individual blood samples were collected with 5 mL sterile heparin tubes. Immediately after recovery, blood samples were centrifuged at 1,500×*g* for 15 min and the plasma was separated and biobanked into polypropylene vials at − 20 °C until the analysis of metabolic biomarkers (glycemic values and lipids profile). Glucose and fructosamine (parameters related to glucose profile), as well as parameters related to lipid profile total cholesterol, high-density lipoprotein cholesterol (HDL-c), low-density lipoprotein cholesterol (LDL-c) and triglycerides, were measured in plasma. Fructosamine is considered a better index than glucose itself for long periods since it represents average glucose values during previous days [24]. Assays were performed with a clinical chemistry analyzer (Saturno 300 plus, Crony Instruments s.r.l., Rome, Italy), according to the manufacturer’s instructions. The data of kits (SPINREACT, Sant Esteve de Bas, Spain) and reliability criteria for all assays were included in Additional File 2.

### Evaluation of fatty acid composition of diets

Dietary FA were extracted and methylated by the one-step procedure of Sukhija et al. [25]. Fatty acid methyl esters (FAME) were analyzed and identified by gas chromatography (Hewlett Packard HP-6890, USA) with a flame ionization detector and a capillary column (HP-Innowax, 30 m × 0.32 mm i.d. and 0.25 μm polyethylene glycol-film thickness) with a temperature program of 170 to 245 °C as previously described [26]. Results were expressed as gram per 100 g of detected FAME.

### Evaluation of the fat content and fatty acid composition of tissue samples

The procedure of fat extraction was different for backfat samples and liver and muscle samples. In brief, subcutaneous fat was extracted after being separated in outer and inner layers. Each layer was studied separately because the inner layer is metabolically more active than the outer layer, mainly due to a high lipoprotein lipase activity [27]. In the case of liver and LD muscle, samples were previously freeze-dried for three days in a lyophilizer (Lyoquest, Spain) and ground in a Mixer Mill MM400 (Retsch Technology, Germany) until tissues were completely powdered. Liver and LD lipids were extracted as described by Segura et al. [28] and fat tissue was calculated and expressed as a percentage. Afterward, lipids of liver and LD were separated in neutral and polar lipids (NL and PL, respectively) by using aminopropyl minicolumns accordingly with the method employed by Ruiz et al. [29] methylated with pentadecanoic acid (C15:0; Sigma, Spain) as the internal standard [30] and analyzed by gas chromatography as described by Lopez-Bote et al. [26]. The percentages of individual fatty acids were used to calculate proportions of saturated (SFA), monounsaturated (MUFA) and polyunsaturated (PUFA) fatty acids, as well as total n-3 and n-6 and their ratio (Σn-6/Σn-3). The activity of stearoyl-CoA desaturase enzyme 1 (SCD1) was estimated as C18:1/C18:0, while ratios for MUFA/SFA and unsaturation were determined [31, 32].

### Statistical analysis

Data were analyzed using the general linear model (GLM) procedure contained in the SAS version 9.4 (Statistical Analysis System Institute Inc., USA). The model included nutritional status of the sow (control/underfed), BW classification (LBW/NBW) and sex (female/male) as the main effects. Interactions of two-way (BW classification × Nutritional status and BW classification × sex) and the three-way interaction of main effects were examined and showed in tables. Statistical analysis of birth and weaning were blocking for sow to account for the common maternal environment. Litter size (LS), which was categorized into three groups (3–6 piglets/litter, 7–9 piglets/litter, and 10–13 piglets/litter), was used as a random effect for birth data. For performance parameters, age was used as a covariate. Moreover, Duncan’s test was used to identify differences between groups. Chi-square was used to assess the mortality data and the percentage of births by the litter size classified into three groups, as described above. The piglet was the experimental unit for all the variables studied except for the reproductive parameters where sow was the unit, and for the FCR data with the pen as experimental unit. All the results were expressed as mean ± RMSE (root mean square error) in tables, but mean ± SD (standard deviation) was used for reproductive parameters, figures and text. Statistical significance was accepted from *P* < 0.05 and trend was defined between *P*values of 0.05 and 0.10.

## Results

### Effects of maternal undernutrition on prolificacy and litter size

At farrowing, there were not significant differences between control and underfed groups in the total number of piglets born/litter (8.9 ± 2.6 vs. 8.5 ± 2.0 for control and underfed sows, respectively) or piglets born live/litter (8.6 ± 2.6 vs. 8.0 ± 1.8 piglets for control and underfed sows, respectively). Concomitantly, at weaning, there were no significant differences in the mean total number of weaned piglets/litter (7.7 ± 0.7 vs. 7.4 ± 0.7 piglets for control and underfed sows, respectively) or in the total litter-weight/litter (42.1 ± 7.6 kg vs. 41 ± 7.5 kg for control and underfed sows, respectively).

Despite the absence of significant differences in the mean number of piglets/litter, the analysis of LS showed a similar percentage of litters with 3–6 piglets in control and underfed sows (17 vs. 15.2%, respectively) but suggestive differences in the distribution of litters with more than seven piglets. Control sows showed 34.0% of medium litters with 7–9 piglets and 48.9% of large litters with 10–13 piglets, while underfed sows had 54.6% and 30.3% for medium and large, respectively. There were trends for a lower percentage of litters with 7–9 piglets (*P* = 0.07) and a higher percentage of litters with 10–13 piglets (*P* = 0.09) in the control group than in the underfed group.

### Effects of maternal undernutrition and offspring sex on characteristics of piglets at birth

The nutritional status of the sow during pregnancy did not have significant effects on birth weight and most of the body measures of piglets, except control piglets showing larger occipito-nasal length, shorter trunk length and higher thoracic diameter than underfed counterparts (Additional File 3: Table S2). The incidence of LBW piglets was directly related to LS in both groups (*P* < 0.001), being higher in control than in underfed sows (15.8% control vs. 8.4% underfed; *P* < 0.005). The distribution of this incidence was 1.5% in litters of low LS (2.4 vs. 0% for control and underfed litters, respectively), 7.8% in medium litters (5.7 vs. 9.7%, respectively) and 19% in litters with more than nine piglets (23.2 vs. 8.8%, respectively). As expected, weight was higher in NBW than in LBW piglets at birth (1.4 ± 0.2 vs. 0.8 ± 0.2 kg, respectively) and weaning (5.6 ± 1.2 vs. 4.3 ± 1.1 kg; *P* < 0.0001 for both). Occipito-nasal and trunk lengths and abdominal and thoracic circumferences were also greater at birth and weaning in NBW (birth: 12.6 ± 0.6, 23.5 ± 1.8, 19.0 ± 1.6 and 24.3 ± 1.5 cm and weaning: 16.2 ± 0.9, 40.1 ± 3.8, 33.4 ± 3.7 and 38.5 ± 3.3 cm, respectively) than in LBW piglets (birth: 11.7 ± 0.6, 19.6 ± 1.6, 15.4 ± 1.6 and 19.8 ± 1.9 cm and weaning: 15.3 ± 1.1, 35.6 ± 4.1, 30.5 ± 3.1 and 35.0 ± 3.6 cm, respectively; *P* < 0.0005 for all). There were no significant effects of sex on the total incidence of LBW piglets, being 14.5% for males and 11% for females. On the other hand, there were sex-related effects on the thoracic circumference at birth; such value was greater in females than in males (23.9 ± 2.0 vs. 23.5 ± 2.3 cm, respectively; *P* < 0.01) and LBW-males showed the smallest circumference (19.4 ± 2.0 cm; *P* < 0.05 for the interaction effect).

### Assessment of early-postnatal development (suckling phase)

**Fig. 2:**
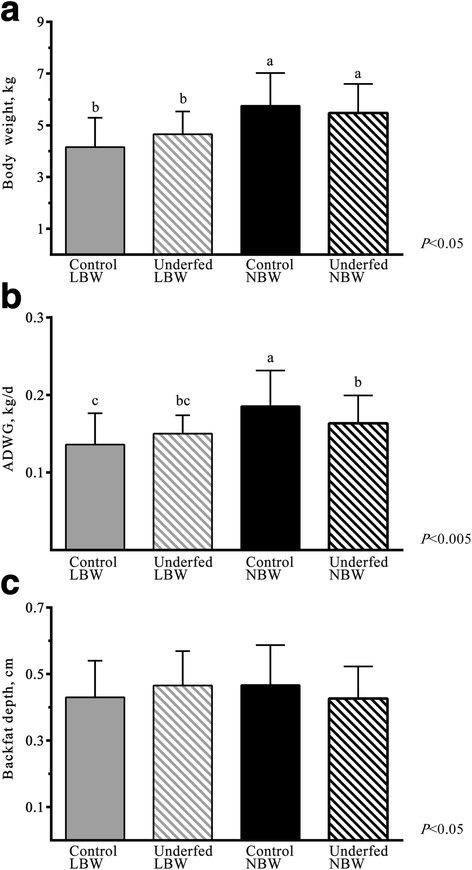
Effect of birth-weight and nutritional status of sows at weaning. Mean ± SD values of body weight (**a**), ADWG (**b**) and backfat depth (**c**) in control LBW, underfed LBW, control NBW and underfed NBW pigs at 24 days old. Different letters indicate significant differences by Duncan’s test. LBW = Low birth weight, NBW = Normal birth weight, ADWG = Average daily weight gain

The analysis of ADWG during lactation and weight at weaning showed an interaction between maternal diet and BW (Fig. 2 and Additional File 3: Table S2). The NBW piglets of the control group showed higher ADWG and weight than the NBW piglets from underfed sows. The effect was the opposite in the LBW piglets since LBW piglets of the underfed group had higher ADWG and weight than LBW piglets of the control group (Fig. 2; *P* < 0.005 and *P* < 0.05 for the interaction effect, respectively). These effects were also found when assessing the backfat depth, which was higher in underfed-LBW and control-NBW groups than in the remaining piglets (Fig. 2; *P* < 0.05 for the interaction effect). Conversely, such interaction was not found in the values for loin diameter, and NBW and control piglets (0.9 ± 0.3 and 1.0 ± 0.2 cm) had higher values than LBW and underfed piglets (0.8 ± 0.3 and 0.8 ± 0.2 cm) respectively (*P* < 0.0001 for birth weight and *P* < 0.01 for maternal diet). The assessment of body size showed the largest occipito-nasal and trunk lengths and abdominal and thoracic circumferences in the control-NBW piglets (16.3 ± 1.0, 40.2 ± 4.1, 34.4 ± 3.4 and 38.9 ± 3.5 cm, respectively) and the smallest in the control-LBW piglets (15.2 ± 1.2, 35.0 ± 4.5, 30.4 ± 3.4 and 34.7 ± 3.9 cm, respectively; *P* < 0.05 for the interaction effect). Moreover, the abdominal perimeter was greater in the control group than in the underfed group (33.9 ± 4.0 vs. 31.9 ± 2.8 cm, respectively; *P* < 0.05).

### Assessment of late-postnatal development (growing-fattening phase)

It is noteworthy that, during the growing-fattening phase, mortality was higher in the underfed group than in the control group (6.5% control group vs. 3.3% control group; *P* < 0.05), especially in the period from 215 days old to slaughter.

**Fig. 3:**
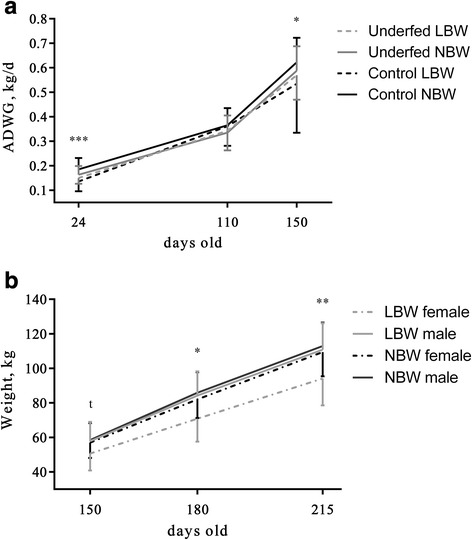
ADWG and weight during the growing phase. Mean ± SD values of ADWG (**a**) and body weight (**b**). Panel A: control LBW, underfed LBW, control NBW and underfed NBW pigs. Panel B: LBW female, LBW male, NBW female and NBW male pigs. LBW = Low birth weight, NBW = Normal birth weight, ADWG = Average daily weight gain. Asterisks indicate significant differences between groups (*t* = 0.1 > *P* > 0.05, *P < 0.05, **P < 0.01, ***P < 0.005)

The maternal diet also had a significant influence on body weight, ADWG and FCR. At 110 days old, underfed-LBW-females showed the lowest body weight and ADWG values and the greatest FCR (Additional File 4: Table S3 *P* < 0.005 for the interaction effects) while control-LBW-females had the lowest FCR and the greatest ADWG. However, control-LBW-females showed the highest FCR and the lowest ADWG at 150 days old (*P* < 0.05 for the interaction effects). Afterward, body weight was lower at 180 days old in offspring from controls sows (82.3 ± 11.5 vs. 84.7 ± 12.4 kg; *P* < 0.05), but higher at 215 days old than in offspring from underfed sows (113.6 ± 13.8 vs. 107.2 ± 14.3 kg; *P* < 0.0003; Fig. 3). Furthermore, underfed pigs had also lower ADWG and higher FCR than control pigs at 215 days old (0.7 ± 0.1 vs. 0.9 ± 0.1 kg/d and 5.2 ± 1.3 vs. 3.9 ± 0.9 kg/kg; *P* < 0.0001 for both).

Birth-weight also significantly affected values of ADWG, FCR, and body weight. In both control and underfed groups, the values for body weight were always higher in NBW than in LBW pigs (150 days old: 57.7 ± 9.3 vs. 54.7 ± 11.1, 180 days old: 84 ± 11.5 vs. 78.5 ± 15.1 and 215 days old: 111.2 ± 13.9 vs. 103.9 ± 17.4 kg; *P* < 0.05 for all age-periods; Additional File 4 Table S3). From 150 days old onwards, the LBW group showed lower ADWG (150 days old: 0.54 ± 0.2 vs. 0.6 ± 0.1, 180 days old: 0.66 ± 0.2 vs. 0.71 ± 0.1 and 215 days old: 0.75 ± 0.2 vs. 0.82 ± 0.2 kg/d) and higher FCR than the NBW group (150 days old: 2.4 ± 1.2 vs. 2 ± 1.0, 180 days old: 4.5 ± 1.4 vs. 3.7 ± 0.7 and 215 days old: 5 ± 1.6 vs. 4.5 ± 1.2 kg/kg; *P* < 0.005 for all).

Offspring sex showed a significant effect from 150 days old; males had higher body weight, higher ADWG and lower FCR than females at 150 days old(58.4 ± 9.9 vs. 56.5 ± 9.1 kg, 0.62 ± 0.2 vs. 0.58 ± 0.1 kg/d and 2.0 ± 0.9 vs. 2.1 ± 0.9 kg/kg, respectively) and 180 days old (85.7 ± 12.1 vs. 81.2 ± 11.4 kg, 0.8 ± 0.2 vs. 0.7 ± 0.1 kg/d and 3.8 ± 0.8 vs. 3.9 ± 1.0 kg/kg; *P* < 0.01, *P* < 0.0001 and *P* < 0.05 for both periods, respectively). Moreover, LBW-females showed the highest FCR but the lowest ADWG at 180 days old (4.9 ± 1.6 kg/kg and 0.57 ± 0.2 kg/d) and the lowest body weight at 180 and 215 days old (Fig. 3; *P* < 0.05 for the interaction effects).

At slaughter, the assessment of the ADWG for the overall period showed that control, male and NBW pigs had higher ADWG (0.56 ± 0.1, 0.55 ± 0.1 and 0.55 ± 0.1 kg/d, respectively) than their respective counterparts (underfed, female and LBW pigs, 0.53 ± 0.1, 0.54 ± 0.1 and 0.50 ± 0.1 kg/d, respectively; Table 1). The overall ADWG and slaughter weight were the lowest in LBW-females (0.47 ± 0.1 kg/d and 140.1 ± 18.6 kg; *P* < 0.005 for the interaction effects), but they showed the greatest slaughter age (301.6 ± 20.5 days old; *P* < 0.005 for the interaction effect). Regarding BW effect, NBW pigs were slaughtered at a younger age than LBW pigs (277.4 ± 19.6 vs. 292.3 ± 21.1 days old, respectively; *P* < 0.0001). Moreover, control-NBW and -LBW pigs showed the lowest and the highest age to slaughter (271.9 ± 23.4 and 293.8 ± 25.4 days old, respectively; *P* < 0.01 for the interaction effect).

**Table 1:** Carcass and meat quality traits at slaughter

Variables	N	Control								Underfed								RMSE	*P*-value					
		LBW				NBW				LBW				NBW										
		Females		Males		Females		Males		Females		Males		Females		Males			BW	Sex	Nutri	BW× Sex	BW× Nutri	BW×Sex×Nutri
Body weight, kg	442	141.5^c^		151.0^a^		153.4^a^		154.0^a^		137.7^c^		147.1^a,b^		152.0^a^		151.5^a^		9.7	<.0001	0.004	t	0.005	ns	ns
Age, d	442	306.3^a^		284.7^b,c^		269.5^d^		274.3^c,d^		293.1^b^		287.2^b,c^		284.4^b,c^		282.2^b,c,d^		18.9	<.0001	ns	t	0.02	0.01	t
ADWG, kg/d	442	0.47^c^		0.54^a,b^		0.57^a^		0.57^a^		0.47^c^		0.51^b^		0.54^a,b^		0.54^a,b^		0.06	<.0001	0.007	0.04	0.004	ns	ns
Carcass weight, kg	395	118.0^a,b^		121.6^a,b^		122.1^a^		122.2^a^		108.0^c^		115.0^b^		118.8^a,b^		118.4^a,b^		8.1	0.002	ns	0.0001	t	ns	ns
Carcass yield, %	395	79.96^a,b^		80.55^a^		79.26^a,b,c^		79.16^a,b,c^		78.61^c^		78.14^c^		78.13^c^		78.14^c^		1.94	t	ns	<.0001	ns	ns	ns
Carcass length, cm	376	88.10^a,b^		88.23^a,b^		90.55^a^		90.25^a,b^		87.83^b^		87.83^b^		89.51^a,b^		88.80^a,b^		2.66	0.002	ns	ns	ns	ns	ns
Backfat depth, cm	386	5.26^a^		5.29^a^		5.17^a,b^		5.16^a,b^		4.62^b^		4.64^b^		4.90^a,b^		4.80^a,b^		0.70	ns	ns	0.0005	ns	ns	ns
Muscular dry matter, %	386	30.32^a,b^		31.49^a^		29.89^b^		30.56^a,b^		29.66^b^		30.82^a,b^		30.32^a,b^		30.37^a,b^		5.49	ns	0.02	ns	ns	ns	ns
Liver dry matter, %	337	30.44^b^		29.78^b^		29.81^b^		29.71^b^		32.45^a^		32.82^a^		30.52^b^		31.53^a,b^		4.29	0.04	ns	<.0001	ns	ns	ns
Muscular drip loss, %	325	6.92^a^		3.83^b^		5.53^a,b^		5.62^a,b^		5.65^a,b^		4.59^a,b^		6.42^a^		6.34^a^		2.64	ns	t	ns	t	ns	ns
Intramuscular fat, %	387	27.75^a,b^		28.04^a,b^		24.37^a,b^		26.91^a,b^		24.01^a,b^		29.22^a^		22.75^b^		25.78^a,b^		6.45	t	0.03	ns	ns	ns	ns
Liver fat, %	345	18.00^c^		24.46^a^		20.48^b,c^		20.80^b,c^		19.60^b,c^		21.93^a,b^		21.08^b^		20.36^b,c^		2.74	ns	0.004	ns	0.002	ns	t

*BW* Birth weight, *Nutri* Maternal Nutrition, *LBW* Low birth-weight, *NBW* Normal birth-weight, *RMSE* root-mean-square error, *ns* not significant, *t* = 0.1 > *P* > 0.05. Different letters indicate significant differences (*P* < 0.05)

Effects and the interaction of maternal diet, BW and sex were also found when assessing backfat depth; underfed, NBW and male pigs (2.5 ± 0.5, 2.4 ± 0.5 and 2.5 ± 0.5 cm, respectively) had thicker backfat than their respective counterparts at 215 days old (control, LBW and female pigs, 2.3 ± 0.4, 2.2 ± 0.6 and 2.3 ± 0.5 cm respectively; Additional File 4: Table S3). Moreover, at such age, males also had a higher loin diameter than females (3.5 ± 0.7 vs. 2.9 ± 0.6; *P* < 0.0001).

### Evaluation of plasma indexes of glucose and lipids metabolism

**Fig. 4:**
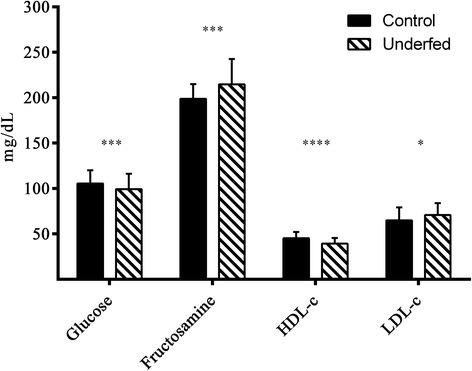
Effect of maternal feed restriction on metabolic status at 215 days old. Mean ± SD values of glucose, fuctosamine, HDL-c and LDL-c concentrations in control and underfed pigs. Asterisks indicate significant differences between groups (*P < 0.05, **P < 0.01, ***P < 0.005, ****P < 0.0001)

The plasma indexes of glucose and lipid metabolism at adulthood were mainly affected by the maternal nutritional status during pregnancy. At 215 days old, the pigs of the underfed group showed lower plasma concentrations of glucose and HDL-c than pigs of the control group (Fig. 4; *P* < 0.005 and < 0.0001, respectively), but higher values for fructosamine and LDL-c (*P* < 0.005 and < 0.05, respectively). At slaughter, a sex-related effect, independently from maternal diet and BW, was observed on triglyceride concentrations, which were higher in females than in males (42.4 ± 12.9 mg/dL vs. 38.9 ± 12.5 mg/dL, respectively; *P* < 0.05).

### Assessment of carcass features and evaluation of drip-loss and moisture

Carcass features, meat quality and characteristics of liver tissue were significantly affected by maternal nutritional status, BW, and sex (Table 1). In brief, control pigs had higher carcass weight and yield (122.0 ± 6.1 vs. 118.1 ± 9.7 kg and 79.3 ± 2.2 vs. 78.1 ± 1.7%; *P* < 0.0001 for both) and higher backfat depth than underfed pigs (5.2 ± 0.7 vs. 4.8 ± 0.7 cm, respectively; *P* < 0.0005). Birth-weight affected carcass weight and length, which were greater in NBW than in LBW pigs (120.3 ± 7.9 vs. 116.5 ± 12.4 kg and 89.7 ± 2.8 vs. 88.0 ± 2.2 cm, respectively; *P* < 0.005 for both), while sex affected muscular dry matter and LD fat content with a higher amount in males than in females (30.5 ± 1.9 vs. 30.1 ± 1.4% and 26.6 ± 6.8 vs. 23.6 ± 6.1%, respectively; *P* < 0.05 for both). Low BW-males also showed the largest liver fat content (22.7 ± 3.8%; *P* < 0.005 for the interaction effect). Moreover, the dry matter of liver samples was lower in NBW (30.5 ± 2.0 vs. 31.7 ± 2.2%, respectively; *P* < 0.05) and control pigs (29.8 ± 0.9 vs. 31.1 ± 2.4%, respectively; *P* < 0.0001) than in LBW and underfed groups, respectively.

### Evaluation of the fatty acid composition of the tissues

Maternal nutritional status and offspring sex were the main factors affecting fatty acid (FA) profile of the different tissues (liver, LD and subcutaneous fat).

In the liver (Additional File 5: Table S4), maternal nutritional status had more effects on FA composition of the Polar Lipid (PL) fraction than on the Neutral Lipid (NL) fraction. The assessment of the PL fraction showed that underfed pigs had greater concentrations of C16:1n-9, C18:1n-9 and MUFA (0.3 ± 0.1 vs. 0.25 ± 0.1, 17.7 ± 1.9 vs. 16.4 ± 2.3 and 20.8 ± 2.3 vs. 19.3 ± 2.7 g/100 g, respectively) and higher desaturase indexes (C18:1/C18:0 and MUFA/SFA; 0.66 ± 0.1 vs. 0.6 ± 0.1 and 0.43 ± 0.1 vs. 0.39 ± 0.1, respectively), but lower concentrations of C22:4n-6 than control pigs (0.7 ± 0.2 vs. 0.8 ± 0.2 g, respectively; *P* < 0.05 for all the values). The composition of PL fraction was also affected by BW, with higher C18:0 concentration in LBW than in NBW pigs (30.6 ± 1.8 vs. 29.5 ± 2.1 g/100 g; *P* < 0.05). There were also sex-related effects since females showed higher C18:2n-6 concentrations and lower C18:3n-3 values than males (12 ± 1.1 vs. 11.7 ± 1.0 and 0.23 ± 0.1 vs. 0.25 ± 0.1 g/100 g, respectively*; P* < 0.05 for both). Moreover, control-LBW-females showed the lowest values of C16:0 and LBW-males had the highest C17:1 levels in this fraction (0.27 ± 0.1 g of C17:1/100 g; *P* < 0.05 for the interaction effects). On the other hand, the assessment of the NL fraction showed higher C20:1n-9 and C20:3n-6 concentrations and ∑n-6/∑n-3 ratio in the control group than in the underfed group (0.7 ± 0.3 vs. 0.4 ± 0.1, 0.5 ± 0.2 vs. 0.4 ± 0.2 g/100 g and 9.4 ± 1.2 vs. 8.3 ± 1.0, respectively; *P* < 0.01 for all). Furthermore, control pigs had lower C18:3n-3 and C22:6n-3 values than underfed individuals (0.3 ± 0.1 vs. 0.4 ± 0.1 and 0.9 ± 0.3 vs. 1.1 ± 0.3 g/100 g, respectively; *P* < 0.05). In the NL fraction, the values of C16:1n-9 and C20:3n-6 showed a triple interaction (*P* < 0.05 for both). No sex effects were found.

In LD muscle (Additional File 6: Table S5), main differences were also observed in the PL fraction due to the influence of maternal diet and offspring BW. The assessment of the PL fraction showed higher concentrations of C18:1n-9, n-3 FA, MUFA, and C20:1n-9 (15.4 ± 2.3 vs. 13.5 ± 1.5, 3.6 ± 0.5 vs. 3.1 ± 0.2, 21.6 ± 2.8 vs. 21 ± 1.8 and 0.33 ± 0.1 vs. 0.26 ± 0.1 g/100 g, respectively), lower levels of C16:1n-9 and SFA (0.3 ± 0.1 vs. 1.3 ± 0.3 and 31 ± 1.6 vs. 32.9 ± 1.7 g/100 g, respectively) and lower ∑n-6/∑n-3 ratio in control than in underfed pigs (12.4 ± 1.3 vs. 13.7 ± 1.0, respectively; *P* < 0.005 for all). Maternal nutritional status also significantly affected the desaturase indexes in the PL fraction, with these indexes being higher in control pigs than in underfed pigs (C18:1/C18:0: 2.1 ± 0.3 vs. 2 ± 0.2 and MUFA/SFA: 0.7 ± 0.1 vs. 0.63 ± 0.1; *P* < 0.05 for both). Birth-weight affected the concentration of PUFA in the control group (47.5 ± 3.5 vs. 46 ± 2.7 g/100 g; *P* < 0.05) and, overall, PUFA levels were higher in control-NBW pigs than in their control-LBW littermates (47.7 ± 3.3 and 45.2 ± 4.0 g/100 g; *P* < 0.01 for the interaction effects). Finally, underfed-LBW pigs had lower C18:0 and C18:1n-9 values than control-LBW (8.3 ± 0.7 and 9.3 ± 0.6 and 13.7 ± 1.5 and 16.8 ± 3.0 g/100 g, respectively) and control-NBW pigs showed the highest C18:2n-6 concentration (29.7 ± 2.2 g/100 g; *P* < 0.05 for the interaction effects). There was also an interaction of maternal diet and BW in values of C16:1n-9, C22:5n-3, MUFA, n-3 FA, n-6 FA and unsaturation index. On the other hand, the assessment of the NL fraction showed higher levels of C16:1n-9, C18:1n-7 and C20:4n-6 and higher ∑n-6/∑n-3 ratio in the NL fraction (0.21 ± 0.0 vs. 0.17 ± 0.0, 4.8 ± 0.6 vs. 3.5 ± 0.6, 0.14 ± 0.1 vs. 0.13 ± 0.1 g/100 g and 4.4 ± 0.8 vs. 4.1 ± 0.7, respectively; *P* < 0.05 for all) in control pigs than underfed pigs. Conversely, control pigs showed lower C18:3n-3 and C18:1n-9 values than underfed pigs (0.46 ± 0.0 vs. 0.5 ± 0.0 and 47.9 ± 2.2 vs. 49.4 ± 2.0 g/100 g; *P* < 0.0001 for both).

In subcutaneous fat (Additional File 7: Table S6), conversely to liver and muscle, the main effects were driven by offspring sex. Males had higher desaturase activity (C18:1/C18:0: inner: 4 ± 0.7 vs. 3.7 ± 0.5 g/100 g and outer: 4.6 ± 0.7 vs. 4.3 ± 0.5; MUFA/SFA: inner: 1.3 ± 0.2 vs. 1.2 ± 0.1 g/100 g and outer: 1.5 ± 0.1 vs. 1.4 ± 0.1) and MUFA concentration than females, both in inner and outer layers (inner: 52.4 ± 2.4 vs. 51.2 ± 1.7 g/100 g and outer: 53.9 ± 2 vs. 52.8 ± 1.7 g/100 g, respectively; *P* < 0.005 for all), and a higher unsaturation index in the inner layer (0.7 ± 0.0 vs. 0.69 ± 0.0, respectively; *P* < 0.01) than females. Conversely, females had greater SFA values at the inner layer (40.6 ± 1.8 vs. 39.4 ± 2.6 g/100 g, respectively; *P* < 0.01) and higher PUFA concentration at the outer layer (9.6 ± 0.9 vs. 9.4 ± 0.7 g/100 g, respectively; *P* < 0.005) than males. Maternal diet also affected FA composition of both inner and outer layers. In the outer layer, underfed pigs had lower SFA values (36.6 ± 1.8 vs. 37.8 ± 2.2 g/100 g, respectively; *P* < 0.0001) and higher MUFA and PUFA concentrations and unsaturation (53.9 ± 1.6 vs. 52.9 ± 2.0, 9.6 ± 0.9 vs. 9.4 ± 0.7 g/100 g and 0.74 ± 0.0 vs. 0.72 ± 0.0, respectively) and desaturase indexes than control pigs (C18:1/C18:0: 4.6 ± 0.6 vs. 4.3 ± 0.6 and MUFA/SFA: 1.5 ± 0.1 vs. 1.4 ± 0.1; *P* < 0.005 for all). In the inner layer, underfed pigs had higher ∑n-6/∑n-3 ratio than control pigs (11.8 ± 4.2 vs. 8.6 ± 0.3, respectively; *P* < 0.0001). Finally, the assessment of possible interactions showed that control-LBW pigs had lower n-6, n-3 and PUFA concentrations (8.5 ± 1.0, 0.63 ± 0.1 and 9.1 ± 0.9, respectively) than underfed-LBW counterparts at the outer layer (9.2 ± 1.1, 0.67 ± 0.1 and 9.9 ± 1.2, respectively; *P* < 0.05, for all the interaction effects). There were triple interactions in the inner layer related to values of C18:1n9, SFA, MFA and unsaturation index and in the outer layer related to C14:0 and C17:0.

## Discussion

The results of the present study indicate that a light maternal feed restriction during mid pregnancy has no effects on sow productivity and piglet phenotype (body weight and size) at birth and weaning. Hence, at first glance, such nutritional management would be adequate for diminishing costs of production. However, the assessment of late postnatal development evidenced that offspring from feed-restricted sows have a higher mortality rate, metabolic disturbances and worse growth patterns with longer time-periods for achieving target-weight and poorer carcass and meat quality traits. Offspring sex and BW modulated these effects, consequently, maternal feed restriction would finally penalize the profitability of the farm.

### Effects of maternal feed restriction on characteristics of litters and piglets at birth and weaning

Maternal feed restriction did not affect the total number of born piglets born nor the mean number of live, stillborn and mummified piglets. Concurrently, there were no differences in birth-weight of piglets from control and underfed groups at farrowing but, on the contrary, there was a lower incidence of LBW piglets in underfed than in control sows.

The higher incidence of LBW piglets in control group may be related to the existence of a higher number of litters with 10–13 piglets in the control group and therefore a greater incidence of LBW in the more prolific litters [3, 4]. The lack of differences in sow productivity and piglet weight may be related to the degree (intake of 70% of the daily requirements) and timing of feed restriction (d 38 to 90 of pregnancy). There are previous studies in lean breeds addressing that a modest reduction in the dietary intake did not affect reproductive parameters of the sows or piglets [2, 33]. Concomitantly, the timing of feed restriction was also determinant for the lack of effects on sow productivity found in the current study. Restriction was started on d 38 of gestation (i.e., after completion of implantation and placentation and beginning of early fetal stage at d 35 of pregnancy, when the most of conceptus losses occurs; [34]) and stopped at d 90 of pregnancy (i.e., early in the third period of gestation, just before fetuses enter the final growth phase when fetal nutrient demand is greatest; [35]). These effects, described in lean breeds, would be even less critical in the Iberian and other traditional breeds due to their high availability of energy stored in fat depots and their ability to mobilize fat depots in the case of undernutrition [9]. The absence of feed restriction during the last days of pregnancy and lactation may also be contributing to the lack of differences in the number of weaned piglets and the total litter weight per sow, since feed intake at the end of pregnancy and the suckling phase are determinant for milk production and composition [36].

However, we have to point out that, despite differences in BW, the nutritional status of the sows during pregnancy affected the morphology of their piglets; piglets from restricted sows had longer trunks at birth and narrower abdomens at weaning. These differences warrant further research since body measures may be interesting to identify piglets with reduced growth potential. In fact, our results may support data addressing that abdominal perimeter may correlate better with postnatal performance than BW, especially during the suckling phase [37].

### Effects of maternal feed restriction and offspring birth-weight and sex on postnatal development

The interaction between maternal nutritional status and offspring birth-weight, modulated by sex, has a prominent effect on postnatal development. At weaning, control-NBW piglets had higher values for body weight, ADWG and backfat depth than the other groups. We have to point out that underfed-LBW piglets evidenced catch-up growth during suckling period, with higher ADWG values than the control-LBW group. However, such catch-up growth was mainly due to a higher fat deposition, which supports previous studies addressing the existence of IUGR effects on appetite-regulation and metabolic pathways [10, 38].

In the growing phase, control-LBW piglets of both sexes caught up with control-NBW pigs. Such pattern of catch-up growth may be related, in this case, to the enrichment of pathways involved in protein deposition and cellular growth [39]. Control-LBW-females had the highest ADWG at 110 days old, despite lowest ADWG at weaning. This catch-up growth could be a mechanism to balance an early low growth. Conversely, underfed-LBW-females showed the lowest ADWG, which reinforces the deleterious effects of maternal feed restriction on offspring development, especially in females.

At later stages, from 150 to 180 days old, males had better growth parameters than females in agreement with data obtained in both modern and traditional breeds [40, 41] and LBW males grew less efficiently than NBW males. In fact, all female groups had lower ADWG than their respective male counterparts; such effect also interacted with BW at slaughter suggesting that both control- and underfed-LBW-female piglets never recovered from a slower growth pattern. Maternal feed restriction also affected growth patterns during the fattening phase. At 215 days old, overall, control pigs showed faster growth and lower FCR than underfed pigs.

Finally, the overall ADWG and the slaughter age was again determined by significant interactions among maternal diet and offspring BW and sex. In brief, pigs from control sows and NBW had better growth-efficiency than their counterparts, in agreement with previous data in lean breeds [11, 42, 43]. Such effects were modulated by sex since males reached heavier final weights, with control and underfed LBW-females showing the lightest values. In conclusion, our results support previous studies addressing more efficient growth patterns in NBW pigs [44, 45].

The differences in growth patterns between groups were concomitant to differences in body composition, in the patterns of muscle development and fat deposition, which may finally affect carcass yield and meat quality. At weaning, underfed and LBW piglets had smaller loin diameters than their counterparts, which may be related to effects from intrauterine restriction, either of maternal or placental origin respectively, on the development of secondary myofibers. The development of these fibers occurs from d 55 to 90 of gestation and it has been described to be modulated by epigenetic nutritional factors [46]; hypothesis reinforced by our results. However, the differences between control and underfed and NBW and LBW piglets were lost afterward, which may indicate a fiber hypertrophy during postnatal growth similar to that described in lean breeds [47, 48]; hypertrophy being stronger in males in all the groups. However, these hypotheses cannot be elucidated with the design of the current trial and further studies are warranted.

Pigs from restricted pregnancies showed a higher trend for adiposity, evidenced by higher values for backfat depth throughout postnatal development, which penalized carcass quality and feed conversion efficiency. Such trend for adiposity is related to the prenatal programming evidenced in these individuals [49] due to a catch-up growth mainly based on fat deposition [50]. Moreover, backfat deposition in underfed pigs were mostly related to a thicker inner layer. Therefore, underfed and control pigs might have different metabolic patterns; a hypothesis confirmed by data from metabolic status at adulthood.

### Effects of maternal undernutrition and offspring birth-weight and sex on metabolic status and carcass and FA composition

The results of the present study supported the existence of differences in both glucose and lipid metabolism between pigs from control and restricted pregnancies. The pigs from restricted pregnancies showed higher concentrations of fructosamine and lower levels of HDL-c but higher of LDL-c (dyslipidemia). These parameters evidence a higher trend for insulin resistance in these individuals, following suboptimal nutrition in utero, catch-up growth and higher fat deposition [38, 51]. Adiposity increases insulin resistance [52] and, in these individuals, higher fructosamine levels evidenced high glucose levels over time while dyslipidemia has also been linked to obesity with insulin resistance [53]. We finally have to highlight a sex-related effect in triglyceride concentrations, which were higher in females. The same effect was previously found in purebred Iberian pigs after prenatal programming [17]. Hypertriglyceridemia may also be indicative of impaired glucose tolerance [54, 55].

The data from carcass traits, concomitantly with other data described above, showed better development in control and NBW pigs during the growing-finishing phase. Control pig had higher carcass yields, despite a lack of significant differences in body weight between control and underfed pigs at slaughter. This may indicate a greater development of internal structures in underfed pigs, such as organs or visceral fat, in agreement with prenatal programming and previous data in purebred Iberian pigs [10, 17].

Data evidencing metabolic disturbances were reinforced by the FA composition analysis of liver. This analysis showed significant effects of nutritional status and mainly, in the PL fraction (i.e., there were more differences in the membrane composition than in storage lipids; [56], which may be related to metabolic disturbances and lipotoxicity. In the PL fraction, concentrations of MUFA and C18:1n-9, which is its main FA, were greater in underfed pigs and, consequently, desaturation indexes were too. These indexes are linked to SCD1 activity indicating an increase of lipogenesis and possibly metabolic disorders [31, 57, 58]. Moreover, a high content of C16:1 (product of SCD1), as we found in underfed pigs, is considered a significant marker of adiposity and insulin dysregulation [59]. Sex-related effects in liver PL fraction were linked to C18 unsaturated FA, highlighting greater C18:2n-6 value and lower C18:3n-3 concentrations in females. Both FA are essential FA and necessary for metabolic processes.

In LD muscle, PL fraction showed more changes due to maternal feed restriction than the storage fraction (NL). Herzberg et al. [60] studied the changes in FA composition that occur during fasting, which mainly focus on the selective mobilization. These authors showed the selective mobilization importance in the storage fraction, whereas in our experiment the main changes occur in the PL fraction with a minor content of C18:0 and n-3 FA in pigs from underfed sows during pregnancy. Moreover, the selective mobilization patterns are different to preserve certain PUFA in special situations as hibernating mammals [61]. However, in our study, underfed pigs showed a lower content of C18:0 and higher content of C18:3n-3 and C18:1n-9 than control pigs in the NL fraction of LD. This FA profile may be related to the content of oxidative muscle fibres [62]. Moreover, these differences could indicate a different mobilization pattern in a light maternal feed restriction during pregnancy than in fasting or hibernating situations.

The analysis of backfat depth, both in the outer and inner layer showed that underfed pigs had a higher desaturation index than controls, which would support previous evidence of metabolic differences in individuals with maternal feed restriction [63]. Moreover, underfed pigs showed greater C18:2n-6 concentrations in the outer layer and lower levels of C18:0 levels in both layers. The levels of C18:2n-6 are significant for the production of quality meat products because high values are associated with rancidity problems and impaired water migration [64].

## Conclusion

In conclusion, the present experiment showed the absence of effects of feed restriction during mid gestation on the total number of piglets born or in the mean BW of the piglets at farrowing. However, the adverse effects of maternal nutritional restriction became evident with increasing offspring age and were related to impaired growth patterns and altered carcass quality and FA composition. Control pigs had better growth patterns and feed conversion efficiency than underfed pigs.

Therefore, maternal nutrition during pregnancy has a critical effect on the productive parameters of their offspring, which is even more important in high-quality production systems with a long finishing phase as used in the Iberian pig and similar traditional breeds.

## Additional files

Additional file 1: Table S1. Calculated analysis (g/kg, dry-matter basis) and fatty acid composition of the diets. (XLSX 12 kb)

Additional file 2: Reliability criteria for biochemical plasma assays. (DOCX 13 kb)

Additional file 3: Table S2. Phenotypic parameters at birth and weaning. (XLSX 13 kb)

Additional file 4: Table S3. Growth during growing-fattening phase. (XLSX 14 kb)

Additional file 5: Table S4. Fatty acids composition of liver (g/100 g total fatty acids). (XLSX 23 kb)

Additional file 6: Table S5. Fatty acids composition of longissimus dorsi muscle (g/100 g total fatty acids). (XLSX 23 kb)

Additional file 7: Table S6. Fatty acids composition of subcutaneous fat (g/100 g total fatty acids). (XLSX 20 kb)

## Abbreviations

ADWG: Average daily weight gain
BW: Birth-weight
FA: Fatty acid
FCR: Feed conversion ratio
HDL-c: High-density lipoprotein cholesterol
IUGR: Intrauterine growth restriction
LBW: Low birth-weight
LD: Longissimus dorsi
LDL-c: Low-density lipoprotein cholesterol
LS: Litter size
MUFA: Monounsaturated fatty acids
NBW: Normal birth-weight
NL: Neutral lipids
PL: Polar lipids
PUFA: Polyunsaturated fatty acids
RMSE: Root mean square error
SCD1: Stearoyl-CoA desaturase enzyme 1
SD: Standard deviation
SFA: Saturated fatty acids
