# Supplementary material for: Maternal undernutrition and offspring sex determine birth-weight, postnatal development and meat characteristics in traditional swine breeds
Source: J Anim Sci Biotechnol. 2018 Mar 19;9:27. doi: 10.1186/s40104-018-0240-6 (PMC5858148; doi:10.1186/s40104-018-0240-6)
Supplement: Supplementary file 2 — Reliability criteria for biochemical plasma assays. (DOCX 13 kb) [file 40104_2018_240_MOESM2_ESM.docx]

|  | **Glucose** | **Fructosamine** | **Triglycerides** | **Total cholesterol** | **HDL-c** | **LDL-c** |
| --- | --- | --- | --- | --- | --- | --- |
| **Kit reference** | 41011 | 1001158 | 41033 | 41021 | MI1001096 | MD41023 |
|  |  |  |  |  |  |  |
| **According to manufacturer’s instructions** |  |  |  |  |  |  |
| Detection limit | 0.3709 mg/dL | 1 µmol/L | 0.000 mg/dL | 0.000 mg/dL | 3 mg/dL | 3.7 mg/dL |
| CV Intra-assay, % | 0.44 | 1.79 | 0.52 | 0.77 | 2.55 | 2.24 |
| CV Inter-assay , % | 2.78 | 1.99 | 3.45 | 2.54 | 4.16 | 3.97 |
| HDL-c: high-density lipoprotein cholesterol, LDL-c: low-density lipoprotein cholesterol. | | | | | | |

**Reliability criteria for biochemical plasma assays.**
